# Liver Biliary Function Evaluation on a 1.5T Magnetic Resonance Imaging Scan by T1 Reduction Rate Assessment Using Variable-Flip-Angle Sequences

**DOI:** 10.1097/RCT.0000000000001582

**Published:** 2024-02-12

**Authors:** Marco Di Stasio, Cesare Cordopatri, Cosimo Nardi, Simone Busoni, Linhsia Noferini, Stefano Colagrande, Linda Calistri

**Affiliations:** From the ∗ Department of Experimental and Clinical Biomedical Sciences, University of Florence–Azienda Ospedaliero-Universitaria Careggi; † Department of Health Physics, UOC Fisica Sanitaria, Azienda Ospedaliero–Universitaria Careggi, Florence, Italy.

**Keywords:** Gd-EOB-DTPA, reduction rate (RR), T1 mapping, variable flip angle (VFA)

## Abstract

**Objective:**

Magnetic resonance (MR) relaxometry is an absolute and reproducible quantitative method, compared with signal intensity for the evaluation of liver biliary function. This is obtainable by the T1 reduction rate (T1RR), as it carries a smaller systematic error than the pre/post contrast agent T1 measurement. We aimed to develop and test an MR T1 relaxometry tool tailored for the evaluation of liver T1RR after gadolinium ethoxybenzyl-diethylenetriaminepentaacetic acid administration on 1.5T MR.

**Methods:**

In vitro/vivo (liver) T1RR values with two 3D FLASH variable-flip-angle sequences were calculated by a MATLAB algorithm. In vitro measurements were done by 2 physicists, in consensus. The prospective in vivo study was approved by the local ethical committee and performed on 13 normal/26 cirrhotic livers. A supplemental test in 5 normal/5 cirrhotic livers, out of the studied series, was done to compare the results of our method (without B1 inhomogeneity correction) and those of a standardized commercial tool (with B1 inhomogeneity correction). All in vivo evaluations were performed by 2 radiologists with 7 years of experience in abdominal imaging. Open-source Java-based software ImageJ was used to draw the free-hand regions of interest on liver section and for the measurement of hepatic T1RR values. The T1RR values of each group of patients were compared to assess statistically significant differences. All statistical analyses were performed with IBM-SPSS Statistics. In vivo evaluations, the intrareader and interreader reliability was assessed by intraclass correlation coefficient.

**Results:**

Our method showed good accuracy in evaluating in vitro T1RR with a maximum percentage error of 9% (constant at various time points) with T1 values in the 200- to 1400-millisecond range. In vivo, a high concordance between the T1RR evaluated with the proposed method and that calculated from the standardized commercial software was verified (*P* < 0.05). The median T1RRs were 74.8, 67.9, and 52.1 for the normal liver, Child-Pugh A, and Child-Pugh B cirrhotic groups, respectively. A very good agreement was found, both within intrareader and interreader reliability, with intraclass correlation coefficient values ranging from 0.88 to 0.95 and from 0.85 to 0.90, respectively.

**Conclusions:**

The proposed method allowed accurate reliable in vitro/vivo T1RR assessment evaluation of the liver biliary function after gadolinium ethoxybenzyl-diethylenetriaminepentaacetic acid administration.

Liver function estimation is difficult and inaccurate, and the use of Indocyanine Green Clearance is inadequate to properly assess the “hepatic functional reserve,” even when used in cooperation with techniques as Fibroscan, Child-Pugh classification, and Mayo End Stage Liver Disease score.^1^ Functional hepatic evaluation is helpful in patients with chronic liver disease, as it allows to calculate the appropriate timing of transplantation, and it is crucial to prevent postoperative hepatic failure after extensive liver resection. Multiple targets for antifibrotic agents have also been identified, but the development of these treatments was hindered by the relative lack of a sensitive and specific biomarker that could quantify fibrosis variations.^1^ The hepatocyte-specific uptake of gadolinium ethoxybenzyl-diethylenetriaminepentaacetic acid (EOB) is supposed to be mediated by active membrane transport systems, such as OATP1 and MRP 2, set at the sinusoidal membrane and at the canalicular membrane of hepatocytes, respectively.^2^ EOB-enhanced magnetic resonance imaging (MRI) showed potential efficacy as an alternative evaluation tool since contrast agent (CA) uptake is reduced in cirrhotic livers^2–6^: this results in lower parenchymal signal intensity (SI) in the hepatobiliary phase compared with the healthy liver. However, SI may be influenced by multiple factors including the pulse sequence.^7,8^

Magnetic resonance (MR) relaxometry studies demonstrated that T1mapping could be an absolute and reproducible quantitative index compared with SI.^9^ Because EOB uptake might be a proxy of the liver function, we chose to study the liver T1 reduction rate (T1RR) as defined in the following section. Compared with pre/post-EOB T1 values, T1RR better discriminates between different grades of liver function loss, solely estimating the liver EOB uptake.^6,9,10^ It should be underlined that not all MR scanners are factory equipped with software for T1 mapping evaluation, even though commercial packages for T1 measurement are available for purchase.

On this background, our note aims to propose an MR T1 relaxometry tool tailored to the EOB-enhanced imaging of the liver on a 1.5T scan: specifically, at designing and testing proper MRI sequences that can be processed by a homemade free MATLAB code for an offline T1RR calculation.

## MATERIALS AND METHODS

### Study Population for In Vivo Evaluations

This study was approved by the local ethical committee (OSS 16.264). Our prospective study enrolled 39 patients (24 males and 15 females) from the department of gastroenterology who underwent an MR examination with EOB administration at our institution from 2021 to the end of 2022 for focal liver lesion detection/characterization.

All patients gave their informed consent to undergo computed tomography and MR examinations and participate this protocol. The inclusion criteria were as follows: age between 18 and 80 years, body mass index between 18 and 25 kg/m^2^, and small focal liver lesions (<2 cm) to characterize.

Patients undergoing previous hepatic surgery, regional transarterial chemoembolization or radiofrequency ablation, and patients receiving radiotherapy or chemotherapy at the time of the examination were excluded. Other exclusion criteria were as follows: biliary obstruction, contraindications to the use of gadolinium chelate CA (known hypersensitivity), pregnancy, severe chronic renal failure (glomerular filtration rate <30 mL/min/1.73 m^2^), and common contraindications to MR examinations, such as presence of pacemaker or other medical devices not tested at 1.5T. Liver cirrhosis diagnosis was based on standard clinical, imaging criteria and Fibroscan results.^11^

Patients were stratified using Child-Pugh classification assessed at the same time point of the MR examination,^7,12^ as follows: normal liver (13 patients), Child-Pugh A (14 patients), and Child-Pugh B (12 patients).

Lastly, because the Siemens software for the T1 calculation has been implemented from January 2023 on the MR of our Institution, we have used it for our study. Therefore, to compare the results of our method with those of one standardized commercial tool, we have done a test in a supplemental sample of 10 patients (5 with normal and 5 with cirrhotic liver), out of the studied series, but with the same criteria of enrollment.

### Acquisition

We assessed in vitro and in vivo liver T1 values with two 3D FLASH variable-flip-angle (VFA) sequences^13^ (Table 1) on a 1.5T scan (Aera; Siemens Medical Systems, Erlangen, Germany) with maximum gradient strength of 45 mT/m and peak slew rate of 200 mT/m/ms. Each VFA sequence lasts around 15 seconds.

**TABLE 1:** 3D FLASH Sequences Parameters

Slice thickness, mm	10
Repetition time, ms	5
Echo time, ms	2
No. averages	5
Echo no.	1
No. phase encoding steps	187
Field of view, mm, AP-RL	300–420
Percent sampling	90
Percent phase field of view	82.25
Pixel bandwidth, Hz/px	675
Acquisition matrix	256 × 187
Flip angle	Pre-CA 5°/15° Post-CA 5°/20°
No. rows in the image	208
No. columns in the image	256
Voxel volume, mm	1.5 × 1.5 × 10
Pixel size, mm	1.5 × 1.5

To optimize measurement accuracy, we selected the flip angle (FA) values (5/15 degrees pre-CA and 5/20 degrees post-CA); these values were chosen based on the expected pre- (500–900 milliseconds) and post-CA administration (200–400 milliseconds) T1 value of both normal and cirrhotic liver parenchyma.^2,14^ Both the sequences were tested on phantom to investigate their performances.

In vitro, we used 12 tubes with known and certified T1 values (lowest and highest values of 221 and 1385 milliseconds, respectively). In vivo VFA sequences were acquired pre-CA injection and 20 minutes after CA injection, on hepatobiliary phase, in all patients.

### Image Processing

The SI of the image pixels for a determined FA value (*α_i_*) can be converted into intrinsic T1 values with the following equation:


$${\mathrm{SI}}_{i}=M_{0}\times \sin{\mathrm{(\alpha}}_{i})\times \left(1-\varepsilon_{1}\right)/\left[1-\cos{\mathrm{(\alpha}}_{i}{\mathrm{)\times \varepsilon}}_{1}\right]$$ (1)

where ε_1_ = exp(−TR/T1), SI*_i_* is the signal intensity relative to *α_i_*, *M*_0_ is the equilibrium magnetization value (a constant), and TR is the repetition time.

From the pixel signal intensities of 2 images acquired at different FAs, because TR and *α_i_* are known, it is possible to calculate the T1 value map as reported by Cheng and Wright.^13^ For the offline processing of the images, we developed a simple MATLAB (R2017 b release) code to calculate pre- and post-CA administration T1 relaxation times and report them on colorimetric quantitative maps. The software generates a set of pixel by pixel pre- and post-CA administration T1 maps in DICOM format. The applied filter for smoothing image data is a spatial Gaussian filter with kernel 4 pixel × 4 pixel. The T1RR final map is obtained with the following equation:


$$T1\mathrm{RR}\;\left(\%\right)=100\times \left[\left(\text{T1}\mathrm{pre}-\text{T1post}\right)/T1\mathrm{pre}\right]$$ (2)

where T1pre is the T1 value pre-CA administration, T1post is the T1 value post CA administration (on hepatobiliary phase), and MATLAB code is available in Supplemental Material (http://links.lww.com/RCT/A268).

Flip angle error provided by T1 mapping commercial software was used to evaluate the propagation of uncertainties on T1RR on supplemental sample of 10 patients. The software and all the in vitro measurements were done by 2 physicists (S.M. and L.N.) who worked jointly.

### Phantom–In Vitro Study

**FIGURE 1:**
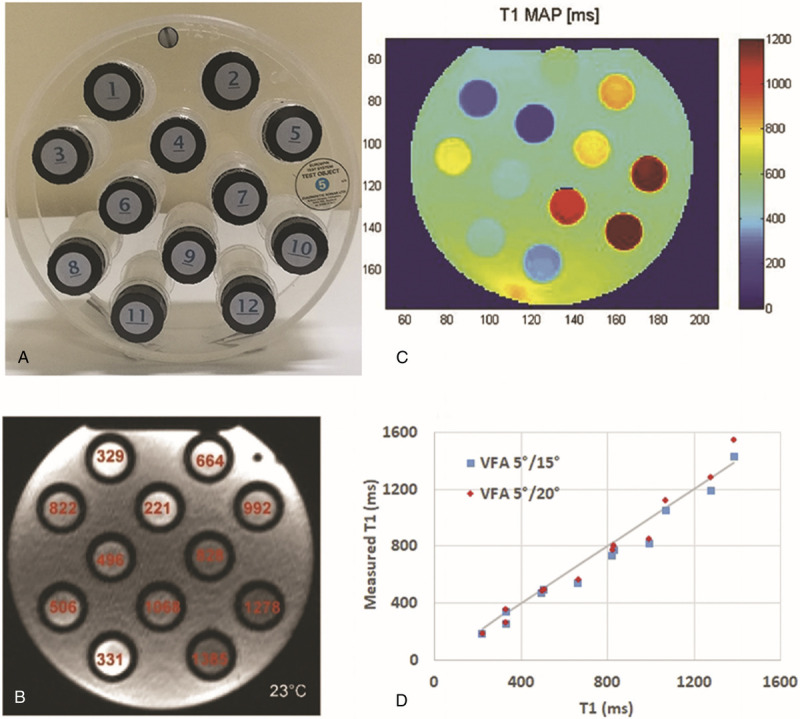
Phantom study. A, The phantom was filled with a paramagnetic solution and had multiple slots for tubes, each one containing a differently doped gel with known and certified T1 at a given temperature. B and C, T1 relaxation times were calculated using a MATLAB-based algorithm and subsequently reported on colorimetric maps. The graphic shows the T1 values obtained with the 2 VFA 3D FLASH sequences (squares: FA 5/15 degrees, diamonds: FA 5/20 degrees) compared with the tubes with certified T1 values (straight line) at time 0 (D).

The sequences were tested on a cylindrical Eurospin phantom (Diagnostic Sonar Ltd, Livingston, Scotland) containing different inserts with known and certified T1 value at a given temperature (Fig. 1). During the study, the phantom temperature was monitored with 0.5°C accuracy, as temperature strongly affects T1 values. We compared the T1 values obtained with the 2 VFA 3D FLASH sequences (FA 5/15 degrees and FA 5/20 degrees) with the tubes with certified T1 values. Test tubes are 20 mm in diameter. Mean T1 value of 3 measurements of a circular region of interest (ROI) with 10 mm in diameter centered on test tube are calculated, so the edge effects due to boxcar filter may be neglected in the ROI. Then, to validate the method, we calculated the T1RR using tubes with known and certified values on the central and peripheral positions of the phantom (Fig. 1). We used 4 tube pairs simulating liver T1 values before and after CA administration (657/325, 1264/487, 657/328, and 813/500). Each pair was measured in 2 slots within the phantom: the pair 652/325 in slots 1 and 12, the pair 1262/487 in slots 2 and 11, the pair 657/328 in slots 4 and 9, and 813/500 in slots 6 and 7. Results are reported hereinafter. The maximum percentage relative error was evaluated with multiple different phantom inserts with the following equation:


$$\Delta \left(\%\right)=\begin{array}{l} 100\times [(T1\mathrm{RR}\;\text{measured}\\ -\;T1\mathrm{RR}\;\text{expected})/T1\mathrm{RR}\;\text{expected}] \end{array}$$ (3)

Repeatability was tested at 15 minutes and 6 hours, with respect to time 0 (8 measures). A new test (5 measures) was repeated at 6 months' intervals, always with respect to time 0.

### In Vivo Study

Patients were asked to fast 4 hours before the test. During the examination, they stretch their arms out over their head and maintain a costal type of breathing (to minimize ghosting and motion artifacts). Three certified test tubes with known T1 value are placed on the patient's abdomen, as additional control in the measurement process, to verify the stability of the temperature conditions during the examination; we accepted only a maximum variation of 5% versus the certified T1 value of each tube between the beginning and the end of the examination. To measure in vivo T1RR, our standard liver MRI protocol was modified by adding the VFA sequences before and after EOB administration (0.025 mmol/kg; Bayer Healthcare, Berlin, Germany) via bolus injection at an infusion rate of 1 mL/s and followed by a 20-mL NaCl flush. The 2 FA images series (FLASH 3D) were acquired in breath-hold (during expiration) for both the pre/post-CA sequences; the pre-CA sequence adopted a 5/15-degree FA, whereas the post-CA sequence, acquired 20 minutes after CA injection, adopted a 5/20-degree FA. Using the aforementioned image processing, a colorimetric T1RR map was generated. Two radiologists with 7 years of experience (M.D.S. and C.C.) in abdominal imaging chose in consensus the slice that showed the best image quality, in the middle portion of the right lobe where artifacts, due to cardiac motion and lung or bowel interfaces, are less likely to happen.^15,16^ Then the T1RR calculation was carried out twice by each reader, independently, with an interval of 1 month, using a PACS workstation (version Syngo plaza-VB30D; Siemens Medical Healthineers, Erlangen, Germany) on the selected slice.

A 2-cm diameter free-hand drawn ROI was positioned in the most homogeneous part of liver parenchyma of the selected slice, at least 2 cm away from the edges of the liver, taking special care to avoid macroscopic vessels and possible focal lesions. Open-source Java-based software ImageJ (1.50i version; https://imagej.nih.gov/ij/) was used to draw the ROIs and to display their T1RR values. The mean T1RR was derived for each patient from the mean of the 3 measurements obtained by each observer. The T1RR values of each group were then compared to assess statistically significant differences.

**FIGURE 2:**
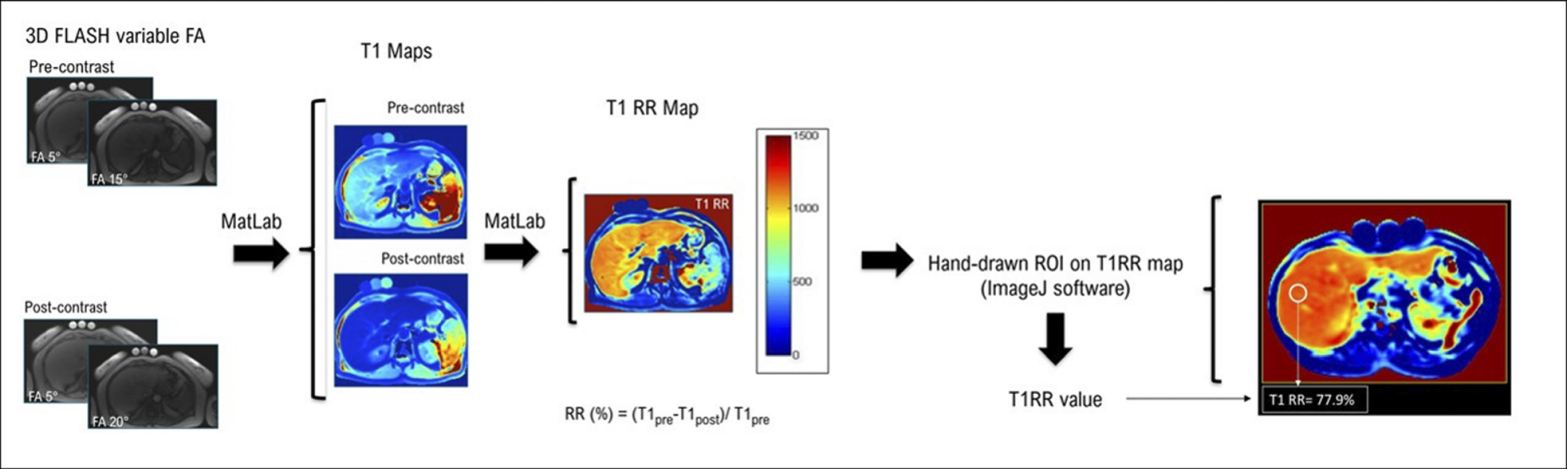
Flowchart of the procedure. To obtain the T1 RR, pre– and post–Gd-EOB-DTPA administration 3D sequences (exported in DICOM format) were acquired, pre– and post–Gd-EOB-DTPA administration T1 maps with the MATLAB algorithm were calculated, and T1 RR map was generated. ImageJ software was used to draw the ROIs and to display their RR values. Three certified test tubes with known T1 value have been placed on the patient's abdomen, as a control in the measurement process, to verify that the T1 RR for the tubes was equal to 0.

Synthetizing procedures to obtain the T1RR map are as follows (Fig. 2):

1) acquisition of pre- and post-EOB administration 3D sequences (exported in DICOM format);
2) pre- and post-EOB administration T1 maps calculation with the MATLAB algorithm; and
3) T1RR map generation using the same software.

A T1 mapping commercial software package (with B1 inhomogeneity correction for VFA methods) was used as criterion standard to evaluate FA errors and T1RR accuracy of our tool (without B1 inhomogeneity correction) in the supplemental sample. The parametric T1 maps were automatically generated on the scanner pre- and post-EOB administration. We followed the same ROI positioning rules, and we calculated the T1RR (%) as explained previously (Equation 2).

### Statistical Analysis

Statistical analyses were performed by IBM SPSS Statistics (version 20; Chicago, IL). Results with *P* values <0.05 were considered statistically significant. Variables were tested for parametric distribution by applying the Shapiro-Wilk test. Normally distributed variables are presented as the mean ± SD. Normality checks showed that the assumption of normality had not been met; thus, the Kruskal-Wallis test followed by the post hoc Dunn-Bonferroni test was the nonparametric test of choice to compare the different patient groups. Nonparametric continuous variables are expressed as the median and interquartile ranges. The intrareader and interreader agreements were determined using the intraclass correlation coefficient (ICC). The ICC values 0.00–0.10, 0.11–0.40, 0.41–0.60, 0.61–0.80, and 0.81–1.0 represented no, slight, fair, good, and very good agreement, respectively. The Bland-Altman test was used to evaluate the concordance between the T1RR evaluated with our method and the Siemens software.

## RESULTS

### Phantom–In Vitro Analysis

First, we tested both the 5/15-degree and the 5/20-degree sequences on phantom; the results are reported in Figure 1. The 2 sequences perform quite similarly over the considered range of T1 values. As expected, the accuracy of the 5/20-degree sequence becomes significantly worse for the highest T1 value (1385 milliseconds).

Our method showed a maximum percentage relative error of 20% in evaluating in vitro T1 values in the 200- to 1400-millisecond range. The main systematic error affecting T1 measurements was space-related. The use of T1RR reduces systematic errors, which are repeated in every T1 measure, such as the error due to inhomogeneity, at least in the first order. This was experimentally confirmed by the multiple T1RR evaluations performed with the tubes in different phantom slots, which allowed accurate results with a maximum percentage error of 9% (Table 2).

**TABLE 2:** Short (15 Minutes) and Medium (6 Hours) Term Measures (*T* = 22.5°C)

Phantom Position (Fig. 1)	A T1 Value, ms	B T1 Value, ms	Expected T1 RR, %	Measured T1 RR, %			Δ (%) (Measured vs Expected T1 RR)		
				0	15 min	6 h	0	15 min	6 h
1	657	325	50.50	50.70	51.00	51.00	1	2	2
2	1264	487	61.50	60.90	60.00	61.80	−1	−2	1
4	657	328	50.10	51.50	51.50	51.20	3	3	2
6	813	500	38.50	41.20	42.20	40.80	6	9	6
7	813	500	38.50	41.90	41.90	41.90	9	9	9
9	657	328	50.10	51.70	51.90	52.20	3	4	4
11	1264	487	61.50	61.20	61.50	62.10	0	0	1
12	657	325	50.50	50.70	50.70	50.70	1	1	1

Δ (%) indicates the percentage error between measured and expected RR values and is calculated by the equation: Δ (%) =100 × ((RR measured − RR expected))/(RR expected). A/B, Phantom tubes with high/low certified T1 values simulating the T1 values of the liver before/after Gd-EOB-DTPA administration, respectively, based on the expected T1 of both normal and cirrhotic liver parenchyma.^2,14^

**FIGURE 3:**
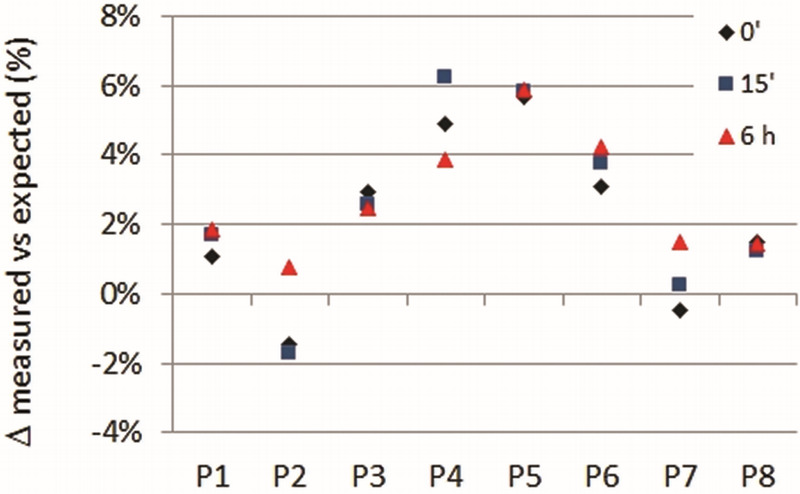
Short- and medium-term percent variations. The percent variations between expected and measured T1 RRs over the short (15 minutes) and medium (6 hours) term measurements (reported in Table 2) are graphically represented. The swapped tube T1s in each phantom position are reported in Table 2. The phantom slot positions (P) are represented on the *x* axis. Diamonds: first measurement (time 0); squares: short-term measurement (15 minutes); triangles: medium-term measurement (6 hours).

The repeatability of the measurements is good: the sequences, tested over the short (15 minutes) and medium terms (6 hours), on the same phantom, showed consistent results with the same maximum percentage error at every different time point (Table 2, Fig. 3). As we can see, the maximum difference between various Δ (%) values in each row (Table 2) is equal to 3%: this represents the actual percentage error between 2 T1RR measures in the same patient at different time points. The test was repeated again at long term (6 months) to check if there were significant differences between cold and hot seasons: the results were completely overlapping, as expected, being the rooms used for the MR device fully air-conditioned.

### In Vivo Study

**FIGURE 4:**
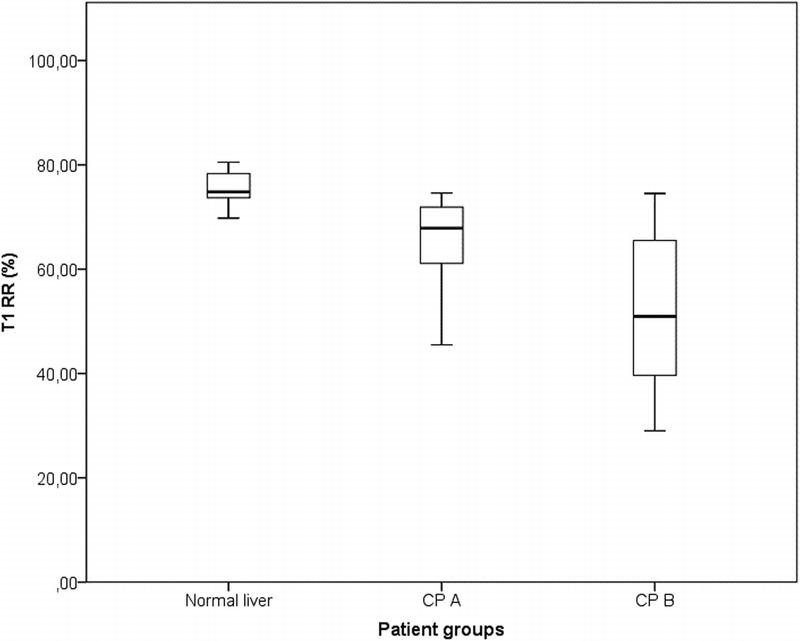
T1 RR values 20 minutes after Gb-EOB-DTPA intravenous administration, stratified by liver function. CP A, Child-Pugh A; CP B, Child-Pugh B. In each box plot, we drew a box from the first quartile to the third quartile. A horizontal line goes through the box at the median. The whiskers go from each quartile to the minimum or maximum.

**FIGURE 5:**
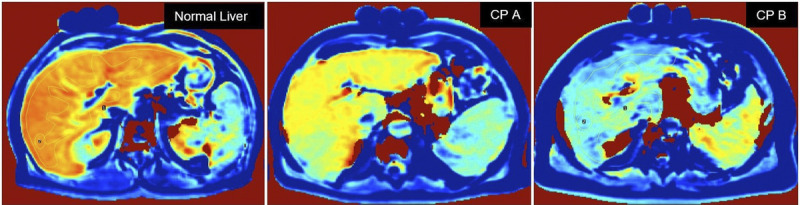
In vivo study. Examples of an RR map in a normal liver and in a Child-Pugh A (CPA) and B (CP B) cirrhosis liver are reported. Certified test tubes with known T1 value are visible on the patient's abdomen, as a control in the measurement process.

A very good agreement was found, both within intrareader and interreader reliability, with ICC values ranging from 0.88 to 0.95 and from 0.85 to 0.90, respectively. Values of T1RR 20 minutes after EOB intravenous administration stratified by liver function are shown (Table 3). A box-and-whisker plot representation of the numerical data is shown (Fig. 4). A T1RR map in a normal liver and in a Child-Pugh A and B cirrhosis liver is reported (Fig. 5). The stability of the T1 values of the tubes positioned over the patients' abdomen as reference during the examination is always maintained (variations <5%).

**TABLE 3:** T1 RR Values Stratified by Liver Function

Liver Function Class	No. Patients	T1 RR, Mean ± SD	Median	IQR
Normal liver	13	75.2 ± 3.4	74.8	72.6–78.5
Child-Pugh A	14	65.8 ± 7.8	67.9	60.8–72.2
Child-Pugh B	12	52.0 ± 15.0	51.0	39.3–65.8

IQR, interquartile range

A Kruskal-Wallis test provided evidence of a statistically significant difference (*χ*^2^(2, n = 39) = 22.2, *P* < 0.001) between the mean ranks of at least one pair of groups, with a mean rank reduction rate (RR) score of 31.38 for the normal liver group, 17.25 for the Child-Pugh A group, and 10.29 for the Child-Pugh B group. Dunn pairwise tests were carried out for the 3 pairs of groups. There was evidence (adjusted using the Bonferroni correction) of a statistically significant difference between the T1RR of the normal liver group and both the Child-Pugh A group (*P* = 0.006) and the Child-Pugh B group (*P* < 0.001).

A high concordance between the T1RR evaluated with our method and that calculated from the Siemens software was verified on 10 patients: the mean differences are normally distributed (*P* = 0.57), with a mean of 0.4% and standard error of 0.9%, and a lower limit of −5.1% and upper limit of 6.0%. No trend is detected on a Bland-Altman plot relatively to the investigated range (60%–80%). The error on the FA, correlated to the B1 inhomogeneities, does not significantly change in the same patient between the measurements done before and after CA administration.

## DISCUSSION

Our homemade MATLAB code for an offline T1RR calculation shows a good in vitro/in vivo accuracy.

The in vitro maximum error is 9% (in the 200- to 1400-millisecond range) with respect to the certified phantom, whereas the in vivo T1RR comparison between the values obtained by ours and a commercial method shows a maximum error of 5%.

The supplemental VFA breath-hold sequences are easy to acquire with an extra time of less than 2 minutes, and the offline T1RR map can be generated in about 5 minutes (Figs. 2, 5). The main systematic error affecting T1 measurements was space-related and, in our opinion, connected to B1 inhomogeneity; this error was not corrected, because for the clinical evaluation, we relied on the T1RR metric with a reduction of the maximum error to 9% (Table 2). However, excluding the values at/out of the reference range of liver T1 values (900 before and 400 milliseconds after CA administration), the maximum error is 6%. The error persisted constantly at the different time points (Table 2, Fig. 3), with a maximum difference between Δ (%) values of around 3%: this could represent the actual percentage error between 2 T1RR measures in the same patient at different time points. Regarding the in vivo study, the T1RR values we found were similar to those obtained with this technique in other studies from the literature,^2,4^ always with T1RR values overlap between various Child-Pugh classes. Haimerl et al^2^ reported T1RR values (mean ± SD) of 65.1 ± 7.1, 57.1 ± 8.8, and 44.3 ± 10.2 for normal liver, Child-Pugh A, and Child-Pugh B patients, respectively. Besa et al^9^ found T1RR values of 67.8 ± 6.6, 59.6 ± 8.5, 45.6 ± 10.0 in the same groups, respectively. Our results indicate that there was evidence of a statistically significant difference between the T1RR of the normal liver group and both the Child-Pugh A group and the Child-Pugh B group. However, as shown, the overlap among the various classes is actually great and so we cannot hope, at present, that a stratification “for individual” is feasible with only this method.

T1 represents a preferable parameter for the evaluation of the liver function and especially the T1RR, as it carries a smaller systematic error.^2^ The reduced EOB uptake may be either due to fewer normal hepatocytes, according to characteristic morphological changes in cirrhotic liver tissue, or to a decreased EOB uptake by hepatocytes, attributed to diminished OATP1 activity.^8,17^ The reference criterion standard for liver fibrosis evaluation is the biopsy, despite a consistent number of limitations (bleeding, discomfort, interobserver/intraobserver variability). However, fibrosis is not a perfectly homogeneous process, and only small hepatic volumes are sampled through biopsy, resulting in the lack of a panoramic view.^18–20^ In the clinical setting, the biopsy is so very often replaced by other less invasive measurement techniques as Fibroscan, APRI, Child-Pugh classification, indocyanine green (ICG) clearance, and Mayo End Stage Liver Disease score.^11,12^

To evaluate liver fibrosis and then, indirectly, liver function, other methods have been adopted, and among these, we remind the last one, that is, the MR elastography.^21^ Although MR elastography is accurate and noninvasive, the application of this technique in routinely practice has been limited by methodological practicability, time required to complete the test (about 20–25 minutes), and hardware cost.^21^ Severe ascites, iron deposition, and high body mass index limit its reliability and, considering chronic liver disease class stratification, an overlap between classes has been demonstrated even using this method.^22^

T1, and so T1RR, can be measured through different techniques such as inversion recovery, look locker, and VFA, which was chosen because we were looking for an accurate but time-saving tool, whereas inversion recovery and look locker are slower.

This study shows how specifically tailored free-of-cost VFA MRI sequences allow effective T1RR calculation. Multiple studies already assessed the utility of T1 mapping before/after EOB administration pointing out the potential of this technique as an MRI-based liver function test.^2,4^ We are aware that many tools are available for such evaluation with MOLLI pulse sequences, currently being the preferred choice,^23^ which is built on an IR acquisition. MOLLI-based T1 mapping tools can be acquired in a breath-hold or free-breathing and are routinely used in cardiac and liver applications. However, a fast volumetric acquisition could be a good choice, and the proposed VFA technique might represent an effective, time-saving alternative for T1RR, which is useful for those who do not have devoted software in the MRI devices available.^9^

We are conscious that the selection of the FAs is always challenging because of 2 main issues.

The first is inaccurate knowledge of the FAs due to transmit field B1 inhomogeneity. Even if the impact on T1 accuracy is known, correction of measured T1 has been rarely reported, mainly because of the complexity and the scarcity of literature and lack of access to vendor-specific B1 mapping sequences.^13,24^

The second source of systematic error is noise-induced bias, with an effect on T1 measurements generally subtle. To address this second problem, various efforts on optimizing the VFA method have been made. The unanimous conclusion is that dual angles were best for achieving the highest efficiency over a narrow T1 range, as we experienced in our study (conversely, multiple angles achieved the most uniform efficiency over a wide T1 range).^13^ As a consequence, the choice of the 2 FAs producing the signal equal to the 71% of SI measured with Ernst angle allows us to optimize accuracy during a single T1 evaluation. T1 accuracy is maximized by choosing the FAs such that SI α1 = SI α2 = 0.71 SI Ernst angle.^13^ The angles to be used before and after the CA administration must be different. In fact, after administration, the T1 shortens and therefore the angle must be modified accordingly (increasing to 5–20 degrees, from 5 to 15 degrees); otherwise, by not taking the variation into account, the measurement would be distorted.

Our study has some limitations. The first is the sensitivity to B1 inhomogeneities: our T1 measurement was affected by a space-related error likely due to this factor, but we did not apply any correction for this issue because we overcome a significant part of it calculating RR, which is what we really needed. Moreover, other authors pointed out that improvement in reproducibility using standard B1 correction methods may be limited, and these are not routinely available on all clinical scanners and therefore of limited use in clinical trials or clinical practice.^25^ Concerning fat and iron content, we know that these affect T1 measurements, but the aim of our study was not to measure and compare the absolute T1 relaxation times of the patients but rather the relative change of T1 relaxation time after EOB administration, which better correlates with liver function.

Another limitation is that we selected only one slice of each liver volumetric acquisition to elaborate T1 and RR maps. For surgical purposes, it could be interesting to expand the RR evaluation to the whole liver volume and to correlate the results with volume measurements. However, this is only a work-in-progress note. In fact, this article is a part of a larger project on the evaluation of liver function funded by the MUR (Italian Ministry of University and Research): the enrollment is ongoing and we aim to improve our method with a whole-liver evaluation.

## CONCLUSIONS

The proposed method allows for accurate and reliable in vitro T1RR assessment and could be used as a cost-effective and non–time-consuming tool for the evaluation of the liver biliary function after EOB administration.

## Supplementary Material

SUPPLEMENTARY MATERIAL
