# Supplementary material for: Liver Biliary Function Evaluation on a 1.5T Magnetic Resonance Imaging Scan by T1 Reduction Rate Assessment Using Variable-Flip-Angle Sequences
Source: J Comput Assist Tomogr. 2024 Feb 12;48(3):354–60. doi: 10.1097/RCT.0000000000001582 (PMC11882171; doi:10.1097/RCT.0000000000001582)
Supplement: SUPPLEMENTARY MATERIAL [file jcat-48-354-s001.pdf]

Dear Author,

Please review the fees to produce your figures in color in print in *Journal of Computer Assisted Tomography*. The price for the first color figure is $750. The charge for each additional color figure is $150.

If this charge meets with your approval, please identify which figures should be printed in color, sign below, and email a copy of this letter to my attention. If your institution or affiliation will cover the cost, please have your purchasing agent sign below. You will be invoiced for all article charges (color separations, reprints, etc.) shortly after your proof edits are returned.

If you do not wish to have your figures print in color, please indicate, “Decline color” below. The figures will appear in black and white in print and in color in the online version of your article. There is no charge for this service.

Prompt return of the signed form, even if you choose to decline color, will avoid delays in publication.

Thank you,

Rio De La Cruz
Proof Manager
Health Learning, Research & Practice
Wolters Kluwer
Email: Rio.DeLaCruz@straive.com

Figures to appear in color: ____

Total cost for color figures: __________________________

Article Number: JCAT-23-273R2

_Linda Calistri___________________________________
Printed Name

_______________________________January 5, 2024___
Signature Date **X Decline Color**
